# Ginsenoside potential targeting hypoxia-inducible factor-1α as promising therapeutics for cancer: a review

**DOI:** 10.3389/fmed.2025.1703982

**Published:** 2026-01-08

**Authors:** Xindan Ai, Anning Li, Ruixue Feng, Hongkang Xu, Hao Yue, Zhe Wang, Yulin Dai

**Affiliations:** 1 Jilin Ginseng Academy, Changchun University of Chinese Medicine, Changchun, China; 2 Jilin Aodong Pharmaceutical Group Co., Ltd., Jilin, China; 3 The Third Clinical Hospital Affiliated to Changchun University of Chinese Medicine, Changchun, China; 4 School of Pharmacology, Changchun University of Chinese Medicine, Changchun, China

**Keywords:** angiogenesis, cancer, epithelial-mesenchymal transition, glycolysis, ginsenoside, hypoxia-inducible factor-1α

## Abstract

Ginseng is a Chinese medicine known for its tonic effect. Numerous studies have shown that ginseng exerts therapeutic effects on cancer cells. Hypoxia-inducible factor-1α (HIF-1α) is an important transcriptional regulator in response to a hypoxic environment and is crucial in the adaptation of tumor cells to the hypoxic environment. During tumor growth, HIF-1α regulates the activity of various transcription factors and their downstream molecules by modulating various biological processes, including cell proliferation, growth, angiogenesis, and metastasis. Therefore, high HIF-1α expression may be closely associated with poor prognosis in patients with various solid tumors. Pharmacological targeting of HIF-1α is considered a therapeutic strategy for cancer treatment. To investigate the molecular mechanism of ginseng’s effect on cancer through HIF-1α, a more detailed and in-depth analysis is needed. In this review, we present ginseng as a HIF-1α inhibitor that can inhibit tumor development *in vivo* and *in vitro* cancer models. These results may clarify the relationship between HIF-1α and ginseng in cancer treatment.

## 1 Introduction

Cancer is a particularly serious disease that threatens the health and lives of people, and its mechanisms of occurrence and therapeutic means have always been a research hotspot in the medical field. Recently, molecularly targeted anticancer drugs have gained widespread attention because of their ability to target specific proteins or mechanisms involved in tumor development, with the advantages of high efficacy, high selectivity, and low toxicity (1). The tumor microenvironment (TME) is the environment that supports tumor growth, comprising various types of cells within the tumor, the tumor vasculature, secreted factors, and the extracellular matrix (2, 3). One distinguishing feature of the TME is hypoxia, where tissue oxygen concentration is below the level required for normal cellular function (4). Hypoxia generates an intratumor oxygen gradient, which contributes to tumor plasticity and heterogeneity, resulting in a more aggressive and metastatic phenotype (5, 6). Hypoxia in TME leads to activation of Hypoxia-inducible factor-1α (HIF-1α), which plays a key role in regulating hypoxia and is a crucial transcription factor that helps cells cope with the hypoxic environment. In the last few years, with the progress in molecular biology and oncology research, HIF-1α involvement in carcinogenesis has gradually attracted attention (7, 8). Furthermore, HIF-1α protein is highly expressed in a variety of solid malignancies, including breast cancer (9), colon cancer (10), gastric cancer (11), lung cancer (12), skin cancer (13), ovarian cancer (14), pancreatic cancer (15), prostate cancer (16), and renal carcinomas (17). Hypoxia can contribute to HIF-1α, which is crucial to helping predict and survive cancer by mediating angiogenesis, glycolysis, and cancer cell invasion and migration (18). Thus, in cancer patients, the expression of HIF-1α is closely linked to the development of many aspects of cancer cell growth, energy metabolism, and metastasis (19). Currently, regulating HIF-1α activity has become an important strategy for the treatment of cancer (20). This regulating is primarily achieved by utilizing chemotherapeutic drugs and small molecule inhibitors that have demonstrated therapeutic efficacy but also face significant challenges in clinical application, including high toxicity and drug resistance (21). In contrast, traditional Chinese botanical drugs are increasingly being recognized for their potential role in cancer therapy, primarily because of their lower toxicity and reduced likelihood of developing drug resistance (22, 23). It is thus of great theoretical significance and practical value to explore the role of Chinese herbs in regulating HIF-1α and its application prospects in cancer treatment.

Research has evidence that the active ingredients in herbs can directly or indirectly regulate HIF-1α expression and activity, inhibit the growth of cancer cells, block angiogenesis, and inhibit the invasion and migration of tumor cells by affecting the stability, transcriptional activity, and its interactions with other transcription factors, thus achieving therapeutic effects on tumors (24–28). Herbs may have an impact on the effectiveness of cancer treatment and reduce side effects through the regulation of the body’s immune function and metabolic pathways (29, 30). *Panax ginseng C. A. Mey.* [Araliaceae] (*P. ginseng*) has been utilized in East Asia for millennia and remains in use today. *P. ginseng* is currently the most widely used medicinal botanical drug worldwide owing to its unique and favorable therapeutic value. In accordance with Donguibogam, a traditional Korean medical text recognized as part of the UNESCO World Heritage, *P. ginseng* has significant tonic properties, enhances intrinsic energy in the body, and has a complementary effect on deficiencies (31). A wide range of ailments are also treated with *P. ginseng*. In a number of modern countries, the potential therapeutic effects of *P. ginseng* tonics have been suggested, and the European Medicinal Plants Committee has also suggested that *P. ginseng* exhibits favorable therapeutic effects on wasting diseases (32). *P. ginseng* is gaining recognition as a promising clinical adjuvant in cancer treatment (33, 34). One of the main active components in this plant is ginsenoside, ginsenosides have been shown to have therapeutic effects against various cancers (35–37), such as gastric cancer (38), breast cancer (39), hepatic cancer (40), thyroid cancer (41), ovarian cancer (42), and colon cancer (43). Research has shown that ginsenosides have preventive and therapeutic effects on cancer through multiple targets, such as phosphatidylinositol 3-kinase (PI3K), protein kinase B (AKT), epidermal growth factor (EGF), HIF-1α, et al.

To the best of our knowledge, no studies provide an exhaustive and comprehensive review of how the components of ginseng exert their therapeutic effects on tumors through the HIF-1α pathway. In this article, the antitumor mechanisms of ginsenosides in hypoxic conditions and their associated metabolic pathways regulated by ginsenosides in response to hypoxia were analyzed. The researchers conducted a literature search across multiple reputable databases, including Web of Science, PubMed, ScienceDirect, and SpringerLink. Using a predefined set of keywords such as “cancer,” “hypoxia,” “HIF-1,” “HIF-1α,” “Warburg effect,” “aerobic glycolysis,” “angiogenesis,” “epithelial-mesenchymal transition,” “ginseng,” and “ginsenoside.” The data presented in this review were collated from relevant articles found through these searches. We believe that this review, which triggers continuous research and exploration of whether other components in *P. ginseng* can play a therapeutic role in cancer through the HIF-1α pathway, will play an important role in cancer treatment.

## 2 Regulation of HIF-1α

**Figure 1:**
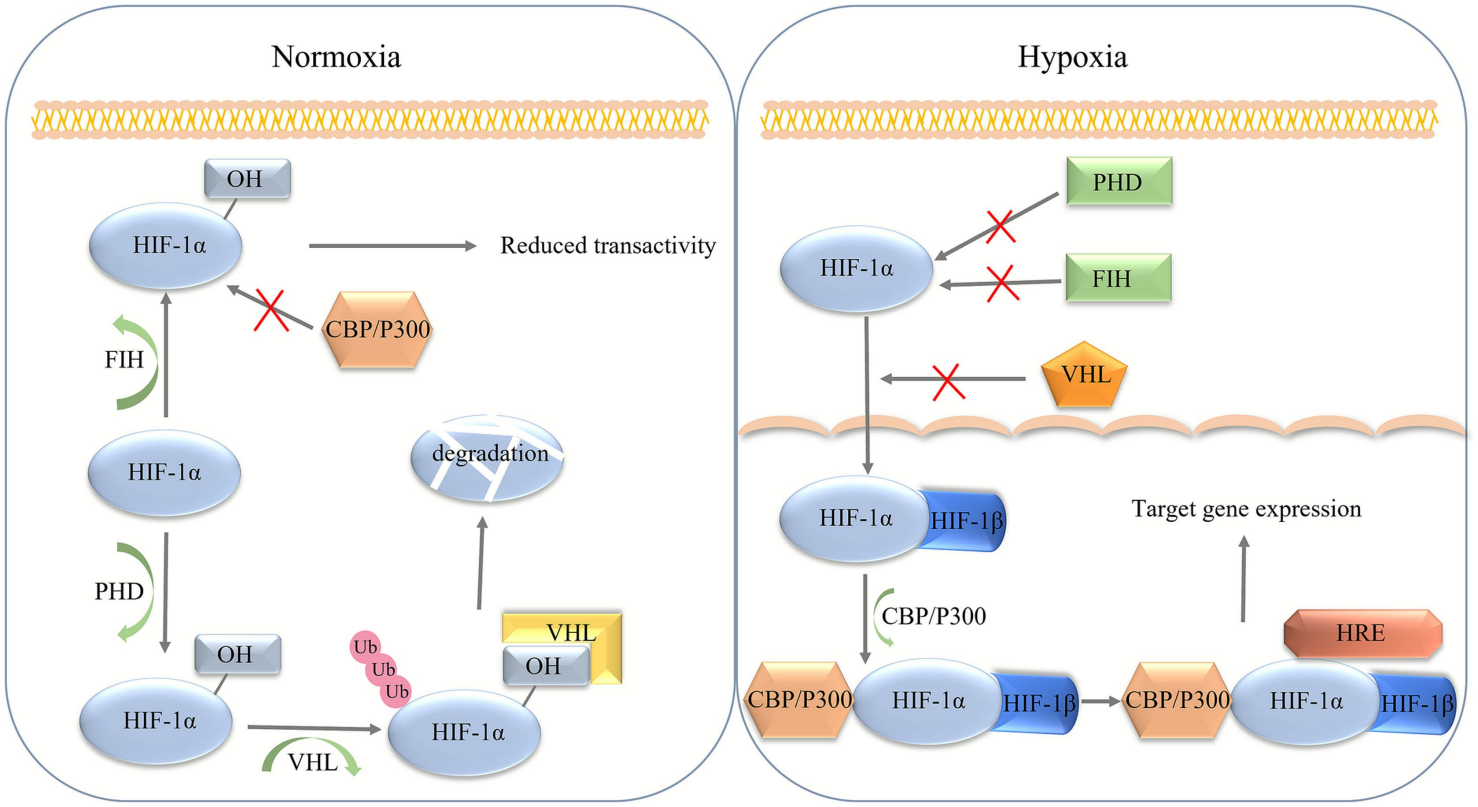
HIF-1α is regulated by oxygen tension. HIF-1α proteins are hydroxylated under normoxic conditions by prolyl hydroxylase domain enzymes. The hydroxylated HIF-1α is then conjugated by the VHL protein, leading to rapid degradation by the proteasome. Additionally, HIF-1α can also be hydroxylated at an asparaginyl residue by the FIH enzyme, which inactivates HIF-1α transcriptional activity by preventing interaction with its transcriptional co-activators. Under conditions of hypoxia, hydroxylation of HIF-1α subunits is suppressed, resulting in protein stabilization, accumulation of HIF-1α, and activation of HIF target gene expression.

Under normal conditions of oxygen, HIF-1α undergoes hydroxylation by HIF proline-4-hydroxylase (PHD) on the HIF-1α subunit oxygen-dependent degradation (ODD) at two conserved proline residues, P402 and P564 (44). Hydroxylation of proline enables direct binding to the von Hippel–Lindau (VHL) protein, a component of the E3 ubiquitin ligase complex that recognizes its substrate, which ubiquitinates HIF-1α and promotes its rapid degradation by the proteasome (44, 45). In addition, under normal oxygen conditions, hydroxylation of the asparagine group of factor inhibiting HIF-1 (FIH) also disrupts important interactions between the HIF-1α subunit and co-activators such as CBP/P300 and prevents HIF-1α transactivation (46). Hypoxic conditions result in the inactivation of PHD and FIH, which inhibit the binding of VHL proteins to HIF-1α. The result is the stabilization of HIF-1α in the cell nucleus, where it binds to hypoxia-inducible factor-1*β* (HIF-1β) and forms active heterodimeric transcription factors in the nucleus (47). In the cell nucleus, HIF-1α/β complexes further bind to hypoxia response elements (HREs) and coordinate their involvement in the transcription of specific genes. The formation of HIF-1α and HIF-1β complexes and their binding capacity to HREs on target sequences depends on the presence of the bHLH–PAS structural domain (48). The specific mechanism is illustrated in Figure 1.

## 3 Role of HIF-1α in cancer

In the TME, the oxygen levels drop significantly because of necrosis, inducing tumor cell death due to deoxygenation. Under these conditions, HIF-1α is activated through various mechanisms. One example is the activation of the PI3K/Akt/mammalian target of rapamycin (mTOR)-mediated HIF-1α pathway, as seen in many solid tumors, including colon cancer (49), prostate cancer (50), and breast cancer (51). The HIF-1α pathway can also be stimulated by growth factors in glioblastoma via MAPK/ERK dependent signaling (52). In many cancer cells, nuclear factor-κB (NF-κB) binding sites are present in the promoter of the HIF-1α gene, allowing NF-κB to up-regulate the expression of HIF-1α (53). Additionally, miRNA can also target the regulation of HIF-1α (54). Under these extreme hypoxic conditions, HIF-1α functions to regulate the expression of several genes through multiple pathways to support tumor cell survival and growth. These genes are involved in critical processes such as angiogenesis, epithelial-mesenchymal transition (EMT), and glycolysis. Such physiological processes are predominantly studied using cell-based experiments, with animal models serving as supplementary tools. To induce HIF-1α expression and stabilization in cell-based studies, hypoxic conditions are commonly simulated by incubating cells at 37 °C in an atmosphere containing 5% CO_2_, 94% N_2_, and 1% O_2_. By promoting these processes, HIF-1α facilitates tumor cell proliferation, invasion, metastasis, and resistance to various therapies, ultimately contributing to cancer progression (55). The following is a detailed description of some of the changes that HIF-1α can induce.

### 3.1 Angiogenesis

Angiogenesis plays an important role in tumor growth, maintenance, and metastasis and is therefore considered a hallmark of cancer progression (56, 57). Blocking angiogenesis is considered a key strategy for inhibiting tumor development. One of the key drivers of angiogenesis is HIF-1α. Several HIF-1α target genes have been shown to be modulators of angiogenesis (58). Angiogenesis is a multifaceted process controlled by numerous positive and negative regulators within the microenvironment (59). Positive regulators include fibroblast growth factors, hepatocyte growth factors such as vascular endothelial growth factors (VEGFs), platelet-derived growth factors, and angiopoietins. Negative regulators include thrombospondin, endostatin, angiostatin, and interferons (60). HIF-1α and VEGF are very closely related, and they induce angiogenesis and promote tumor growth by interacting with the MEK, PI3K/Akt, or FAK signaling pathways, inducing vascular permeability and the expression of genes for cell proliferation (61).

### 3.2 EMT

EMT causes the separation of tumor cells from primary or metastatic lesions and the formation of new secondary metastases (62). Consequently, EMT is fundamental to the process of tumor cell metastasis. Under hypoxic conditions, Tumor cells disrupt the basement membrane in a HIF-1α-dependent manner through initiating a proteolytic cascade (63). Hypoxia promotes EMT, facilitating cancer cell migration and metastasis (64). HIF-1α promotes EMT by directly regulating major EMT-inducing transcription factors (EMT-TFs), such as the Snail and Twist families (65, 66). HIF-1α inhibits E-cadherin transcription by up-regulating its repressors, including ZEB1, ZEB2, transcription factor 3, TWIST, and SNAIL, thereby promoting tumor cell metastasis (67–69). Integrins on tumor cell surfaces recognize hypoxic conditions, resulting in the formation of a fibronectin-rich matrix. When these EMT-TFs are activated, epithelial genes are repressed, and mesenchymal genes are activated, disrupting cell adhesion, and rearranging the cytoskeleton to facilitate cell migration. HIF-1α also indirectly promotes EMT through signaling pathways like Notch and Wnt and epigenetic regulators (70, 71).

### 3.3 Glycolysis

Most cancer cells predominantly utilize aerobic glycolysis as their primary energy source, a process that occurs in the presence of oxygen and promotes tumorigenesis and cancer progression. Under hypoxic conditions, the accelerated glycolysis rate provides valuable metabolites for synthesizing DNA, proteins, and lipids, all of which are essential for supporting rapid tumor proliferation. Thus, targeting tumor glycolysis presents a promising approach for cancer therapy (72, 73). In hypoxic conditions, tumor cells prefer glycolysis as an ATP source (74). HIF-1α, a key regulator of glycolysis, influences metabolic patterns in three main ways: enhancing glucose uptake, suppressing the tricarboxylic acid (TCA) cycle, and modulating enzymes related to glycolysis (75). Under hypoxia conditions, HIF-1α upregulates glucose transporters and glycolytic enzymes through binding to HREs within the promoter regions of genes encoding key glycolytic enzymes, thereby increasing their expression (76, 77). HIF-1α can also induce pyruvate dehydrogenase kinase 1, which limits metabolite entry into the TCA cycle via HIF-1α-dependent induction (78, 79). HIF-1α also promotes the transcription of lactate dehydrogenase-A, an enzyme responsible for catalyzing the conversion of pyruvate into lactic acid, while producing nicotinamide adenosine dinucleotide, a cofactor essential for sustained glycolytic activity (80). Through these mechanisms, HIF-1α promotes glycolysis facilitating increased glucose uptake and metabolism, supporting ATP production, and reducing oxidative metabolism to help meet cellular energy demands under hypoxic conditions. Therefore, HIF-1α may serve as a key molecular target through which ginsenosides exert their antitumor effects by inhibiting angiogenesis, glycolysis, and EMT.

## 4 Relationship between HIF-1α and ginsenosides

**Figure 2:**
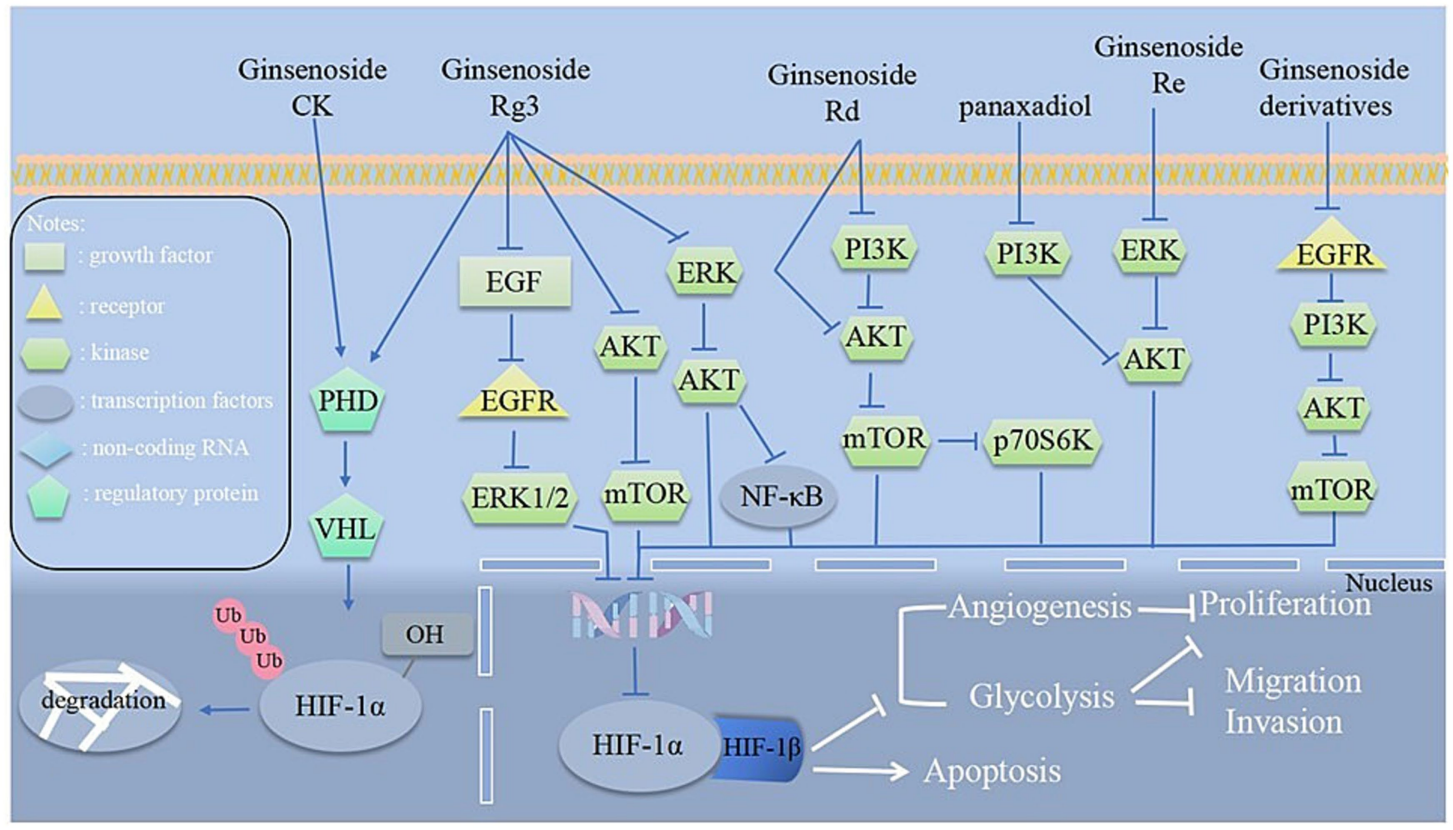
Proposed mechanisms of ginsenosides in modulating HIF-1α pathways. Ginsenosides directly promote the degradation of HIF-1α via the ubiquitin-proteasome pathway and enhance protein expression. Indirect regulation occurs through upstream pathways, including PI3K/Akt/mTOR, MAPK/ERK, and NF-κB signaling, ultimately affecting HIF-1α activity. These mechanisms collectively inhibit cancer progression by modulating HIF-1α through multiple pathways to inhibit angiogenesis and glycolysis of cancer cells.

**Figure 3:**
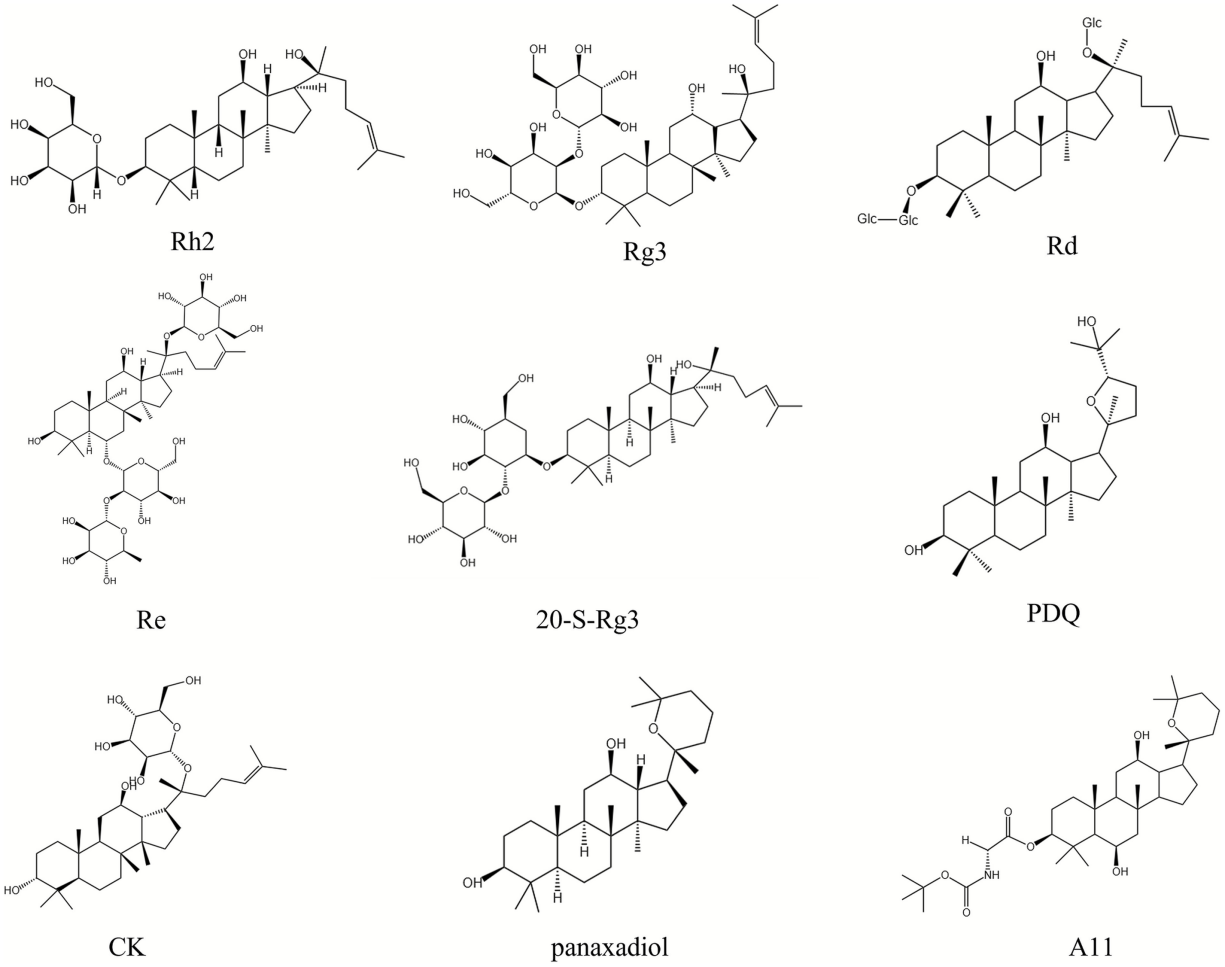
Chemical structure of ginsenoside.

Ginsenosides are the primary bioactive compounds in *P. ginseng*, and has anti-tumor activity through several mechanisms of action. Continuing to research ginsenoside ingredients, the pharmacological activity is becoming a global strategy in the anti-cancer field and is worthy of further investigation (81, 82). Recent studies have demonstrated that ginsenosides exert unique antitumor effects via inhibiting the HIF-1α pathway. They induce apoptosis in tumor cells and suppress tumor growth, proliferation, migration, and invasion, and interfere with angiogenesis. Additionally, ginsenosides have the potential to act as chemotherapeutic adjuvants. When combined with radiotherapy, they can significantly improve drug sensitivity, reverse resistance, regulate metabolism, and provide new avenues for cancer treatment (83–87). The specific mechanisms of the saponin metabolites in *P. ginseng* for cancer treatment via HIF-1α are shown in Table 1 and Figure 2. The chemical structures of all ginsenoside compounds are shown in Figure 3.

**Table 1:** Chemicals targeting the HIF-1α for cancer therapy (↑ increase, ↓ decrease).

Compound	Cancer	Molecular targets	Molecular mechanisms	Experimental model		Reference
				*In vivo*	*In vitro*	
Rg3	hepatocellular carcinoma	EGF↓; EGFR↓; ERK1/2↓; HIF-1α↓	Induction of apoptosis; Inhibition of proliferation	Male BALB/c nude mice	Bel-7402; HCCLM3	(88)
Rg3	breast cancer	Akt↓; HIF-1α↓	Inhibition of proliferation	—	MCF-7; MDA-MB-231	(89)
Rg3	melanoma	AKT↓; ERK↓; HIF-1α↓; VEGF↓	Inhibition of angiogenesis	Male C57BL/6 mice	B16	(90)
20(S)-Rg3	ovarian cancer	PHD↑; VHL↑; HIF-1α↓	Inhibition of EMT and glycolysis	Female nude mice	SKOV3; 3AO	(91)
20(S)-Rg3	gliomas	NF-κB↓; HIF-1α↓	Inhibition of glycolysis	Male nude mouse	T98G; LN229; U87; U251; A172; SVGp12; U373; U118; U138	(92)
Rg3	lung cancer	ERK↓; AKT↓; NF-κB↓; HIF-1α↓	Inhibition of migration and invasion	Male nude mice	A549; SPCA1	(94)
Rg3	osteosarcoma	AKT↓; mTOR↓; HIF-1α↓; VEGF↓	Inhibition of proliferation; Inhibition of metastasis; Inhibition of angiogenesis	Mice receiving 143B xenografts and lung metastases	HUVECs; 143B; U2OS	(95)
Rg3	non-small cell lung cancer	NF-κB↓; HIF-1α↓	Inhibition of EMT	Male nude mice	SPC-A1; H1299; A549	(96)
Rd	breast cancer	Akt↓; mTOR↓; p70S6K↓; HIF-1α↓; VEGF↓	Inhibition of angiogenesis	Sprague–Dawley rats	HUVECs; MDA-MB-231	(97)
Rd	hepatocellular carcinoma	PI3K↓; AKT↓; mTOR↓; HIF-1α↓	Inhibition of proliferation; Induction of apoptosis	Male nude mice	HepG2	(98)
Re	skin cancer	ERK↓; AKT↓; HIF-1α↓	Induction of apoptosis	AB wild-type zebrafish; Male C57BL/6 mice	B16F10	(99)
CK	liver cancer	PHD↑; HIF-1α↓	Inhibition of glycolysis	Clean-grade SD rats	HepG2; SMMC-7721; Bel-7404; Huh7	(100)
CK	non-small cell lung cancer	HIF-1α↓; VEGF↓	Inhibition of proliferation; Inhibition of migration and invasion	Female BALB/c nude mice	H1975; A549; PC9	(101)
Rh2	non-small cell lung cancer	HIF-1α↓	Inhibition of proliferation; Inhibition of glycolysis; Inhibition of migration and invasion	BALB/c nude mice	A549; PC9	(105)
Panaxadiol	colon cancer	PI3K↓; HIF-1α↓	Inhibition of proliferation	Male BALB/c athymic nude mice	HCT116; SW620; HT29; HEK293	(28)
A11	cervical cancer	HIF-1α↓	Inhibition of proliferation; Induction of apoptosis	Male athymic Nu/Nu nude mice	Hep3B; HeLa	(106)
3β-O-Glc-DM	gliomas	EGFR↓; PI3K↓; AKT↓; mTOR↓; HIF-1α↓	Inhibition of proliferation; Induction of apoptosis; Inhibition of angiogenesis	Male Kunming mice	U87 MG; T98G; Hs683; SH-SY5Y; SK-N-SH; G422; GL261	(107)
PDQ	hypopharyngeal cancer	HIF-1α↓	Inhibition of proliferation; Induction of apoptosis	Female BALB/c mice	FaDu	(108)

### 4.1 Ginsenoside Rg3

Ginsenoside Rg3 achieves therapeutic effects on hepatocellular carcinoma by blocking the EGF-epidermal growth factor receptor (EGFR)-extracellular signal-regulated kinase 1/2 (ERK1/2)-HIF-1α signaling axis, blocking the expression of NHE1 (88). Rg3, as a treatment for breast cancer, suppresses the self-renewal of breast cancer stem cells by blocking Akt-mediated HIF-1α activation, thereby eliminating their stemness and inhibiting tumor proliferation (89). In B16 cells, Rg3 inhibits VEGF expression by suppressing HIF-1α, attenuates the proliferation and migration of vascular endothelial cells, as well as reduces melanoma-induced angiogenesis, thereby effectively inhibiting melanoma growth and metastasis (90). 20(S)-Rg3 reduces HIF-1α expression by triggering the ubiquitin-proteasome pathway, promoting HIF-1α degradation. This upregulates the epithelial cell-specific marker E-calmodulin and downregulates mesenchymal stromal cell-specific marker vimentin induced by hypoxia. *In vitro* and *in vivo*, ginsenoside 20(S)-Rg3 effectively inhibited hypoxia-induced EMT and ovarian cancer cell migration and intraperitoneal spreading (91). 20(S)-Rg3 also inhibits HIF-1α expression by attenuating NF-κB expression, reversing the Warburg effect, and stimulating angiogenesis in gliomas (92). Rg3 is also expected to play an adjuvant role in tumor therapy when combined with various cancer treatment drugs. This combination offers strong synergistic potential and enhances therapeutic effects without increasing toxicity (93). Studies provide a biological basis for the clinical evaluation of this approach and offer strong evidence for further research. Gemcitabine (GEM) is a commonly used chemotherapeutic agent for cancer treatment, but its clinical application is restricted due to side effects. Rg3 inhibits NF-κB and HIF-1α nuclear enrichment by eliminating GEM-induced ROS-mediated Akt activation and ERK signaling, thereby reducing GEM-induced ROS production and the migration and invasion of cancer cells (94). Research demonstrates that ginsenoside Rg3 effectively mitigates DOX-induced weight loss and cardiotoxicity in mice, enhances the therapeutic efficacy of DOX in clinical settings, and significantly suppresses cancer cell proliferation, metastasis, and angiogenesis *in vitro* when combined with DOX. Mechanistically, ginsenoside Rg3 and DOX exert antitumor activity by modulating the mTOR/HIF-1α/VEGF and EMT signaling pathways. In addition, in a 143B mouse model of osteosarcoma, ginsenoside Rg3 combined with DOX inhibited tumor growth and metastasis (95). Additionally, the combination of Rg3 and cisplatin increases cisplatin sensitivity and enhances its therapeutic effect on tumors by downregulation of HIF-1α expression and inhibiting tumor cell EMT in hypoxic lung cancer cells (96).

### 4.2 Ginsenoside Rd

Ginsenoside Rd. inhibits tumor angiogenesis *in vitro* and *in vivo* by suppressing HIF-1α/VEGF through the Akt/mTOR/phospho-p70 S6 kinase (p70S6K) signaling pathway, thereby exerting cancer-suppressive effects (97). Furthermore, the combination of Combretastatin A4 phosphate (CA4P) and ginsenoside Rd. exhibits synergistic activity against tumors. Ginsenoside Rd. inhibits HIF-1α protein expression through the PI3K/Akt/mTOR signaling pathway, thereby inhibiting HepG2 cell proliferation and inducing apoptosis both *in vivo* and *in vitro*. CA4P is a vascular disrupting agent that causes rapid occlusion of tumor blood vessels. The combination of CA4P and ginsenoside Rd. improves apoptosis and slows tumor growth (98).

### 4.3 Ginsenoside Re

Ginsenoside Re reduces the expression of Bcl-2 and HIF-1α in tumor xenografts both *in vivo* and *in vitro.* It induces normalization of the tumor vascular system, reduces proliferation, and promotes apoptosis in B16F10 melanomas, exerting an inhibitory effect on the growth of cutaneous melanomas (99).

### 4.4 Ginsenoside CK

Ginsenoside CK has many excellent pharmacological activities as a metabolite of ginsenoside diol ester. Under hypoxic conditions, 20(S)-Ginsenoside CK inhibits Bclaf1 expression and promotes the degradation of HIF-1α by enhancing ubiquitination. In Bel-7404 and Huh7 cells, it inhibits the HIF-1α-mediated glycolytic pathway, thereby suppressing cell proliferation and cancer progression in the TME of hepatocellular carcinoma patients (100). Co-treatment of ginsenoside CK and gefitinib inhibited the angiogenic capacity of HUVEC cells and suppressed the expression of HIF-1α/VEGF, which has a beneficial effect on the normalization of vessel structure. Ginsenoside CK enhanced the anti-proliferative, pro-apoptotic, and anti-migratory effects of gefitinib in primary and acquired drug-resistant non-small cell lung cancer. In conclusion, ginsenoside CK regulates to balance angiogenic factors by down-regulation of the HIF-1α/VEGF signaling pathway and improves the drug resistance of gefitinib, which provides a theoretical basis for the improvement of the clinical efficacy of gefitinib and overcoming drug resistance (101).

### 4.5 Ginsenoside Rh2

Numerous preclinical studies have shown that ginsenoside Rh2 has significant potential for treating a wide range of cancers (102). Ginsenoside Rh2 exerts therapeutic effects on tumors by triggering apoptosis, inducing cell cycle arrest, and suppressing tumor cell proliferation, invasion, and migration (103, 104). Ginsenoside Rh2 inhibits the aerobic glycolysis of tumors, including the uptake of glucose and the production of lactate, by targeting and down-regulating the expression of HIF-1α, which significantly inhibits tumor proliferation and migration (105).

### 4.6 Panaxadiol

Panaxadiol, a monomeric triterpenoid sapogenin found in the roots of *P. ginseng,* was shown by Wang et al. to inhibit hypoxia-induced HIF-1α synthesis via the PI3K and MAPK pathways, without affecting its degradation. Panaxadiol significantly decreased the expression of programmed cell death ligand 1 at both protein and mRNA levels by simultaneously suppressing HIF-1α and STAT3 in a concentration-dependent manner. These results confirm the suppressive effects of panaxadiol on colon cancer cell proliferation (28).

### 4.7 Ginsenoside derivatives

Subsequent studies have found that secondary saponins can be produced by the further hydrolysis of ginsenosides, which also exhibit therapeutic effects on tumors. For example, 20(R)-panaxotriol is a key sapogenin in the ginsenoside family. A11, a derivative of 20(R)-panaxotriol, dose-dependently inhibited the transcriptional activity and protein content of HIF-1α and suppressed nuclear aggregation of HIF-1α in HeLa tumor cells. A11 dose-dependently also inhibits HeLa cell proliferation, promotes apoptosis, and inhibits HeLa cell migration (106). 3β-O-Glc-DM (C3DM) is a biosynthesized ginsenoside that exhibits potent antitumor activity across various cancer cell types, with its *in vivo* anti-colon cancer efficacy surpassing that of ginsenoside 20(R)-Rg3. *In vivo* and *in vitro*, C3DM inhibited EGFR kinase activity, affected the EGFR/PI3K/AKT/mTOR signaling pathway, and significantly inhibited the expression of the HIF-1α protein *in vitro* and *in vivo* protein. C3DM dose-dependently inhibits glioma cell proliferation, invasion, and angiogenesis, induces apoptosis, and significantly inhibits tumor growth in subcutaneous and *in situ* mouse glioma models (107). Pseudoginsengenin DQ (PDQ) is synthesized from the ginsenoside protopanaxadiol, which has good antitumor effects as a secondary ginsenoside. PDQ inhibits FaDu cell proliferation by decreasing glucose uptake and inducing cell cycle arrest and apoptosis. Recent molecular docking studies indicate that PDQ can bind to the active site of HIF-1α in tumors, producing therapeutic effects. In addition, dSTORM analysis revealed that PDQ reduced both the expression and mRNA levels of HIF-1α, inhibited the expression of its downstream effector GLUT1 on the cell membrane, and prevented GLUT1 aggregation. This indicates that PDQ’s antitumor effects are linked to the downregulation of the HIF-1α-GLUT1 pathway, indicating that PDQ may be a potential drug for hypopharyngeal cancer treatment (108).

The research found that attenuated VEGF mRNA levels and hypoxia-induced HIF-1α protein expression were observed in red ginseng-treated HT29 and HCT116 cells. Red ginseng suppressed the mRNA expression of snail, slug, and twist and the protein level of integrin αVβ6. In addition, the hypoxia-induced inhibition of E-calmodulin expression was restored in Red ginseng-treated cells. Red ginseng blocks downstream pathways by inhibiting NF-κB and ERK1/2 phosphorylation in colon cancer cells under hypoxia conditions. Meanwhile, Red ginseng inhibited the hypoxia-induced VEGF expression through the destabilization of the HIF-1α protein, suggesting that Red ginseng may block colon cancer cell invasion and migration by inhibiting these pathways (109). A concise summary of *in vitro* studies on the anticancer effects of ginsenosides is presented in Table 2.

**Table 2:** Summary table of *in vitro* studies on the anti-cancer effects of ginsenosides.

Compound	Cancer	Experimental systems	Effective doses	Main outcomes	Reference
Rg3	hepatocellular carcinoma	Bel-7402; HCCLM3	100 μM	Induction of apoptosis	(88)
Rg3	breast cancer	MCF-7; MDA-MB-231	25 μM	Inhibition of proliferation	(89)
Rg3	melanoma	B16	5 μg/mL	Inhibition of angiogenesis	(90)
20(S)-Rg3	ovarian cancer	SKOV3; 3AO	80 μg/mL; 160 μg/mL	Inhibition of EMT	(91)
20(S)-Rg3	gliomas	T98G; LN229; U87; U251; A172; SVGp12; U373; U118; U138	150 μM	Inhibition of glycolysis	(92)
Rg3	lung cancer	A549; SPCA1	75 μg/mL	Inhibition of migration and invasion	(94)
Rg3	osteosarcoma	HUVECs; 143B; U2OS	160 μg/ml	Inhibition of proliferation; Inhibition of angiogenesis	(95)
Rg3	non-small cell lung cancer	SPC-A1; H1299; A549	200 μg/mL	Inhibition of EMT	(96)
Rd	breast cancer	HUVECs; MDA-MB-231	50 μM	Inhibition of angiogenesis	(97)
Rd	hepatocellular carcinoma	HepG2	20 μM	Inhibition of proliferation; Induction of apoptosis	(98)
Re	skin cancer	B16F10	100 μM	Induction of apoptosis	(99)
CK	liver cancer	HepG2; SMMC-7721; Bel-7404; Huh7	60 μM	Inhibition of glycolysis	(100)
CK	non-small cell lung cancer	H1975; A549; PC9	10 μM	Inhibition of proliferation; Inhibition of migration and invasion	(101)
Rh2	non-small cell lung cancer	A549; PC9	40 μg/mL; 30 μg/mL	Inhibition of proliferation; Inhibition of glycolysis; Inhibition of migration and invasion	(105)
Panaxadiol	colon cancer	HCT116; SW620; HT29; HEK293	10 μM	Inhibition of proliferation	(28)
A11	cervical cancer	Hep3B; HeLa	30 μM	Inhibition of proliferation; Induction of apoptosis	(106)
3β-O-Glc-DM	gliomas	U87 MG; T98G; Hs683; SH-SY5Y; SK-N-SH; G422; GL261	50 μM	Inhibition of proliferation; Induction of apoptosis	(107)
PDQ	hypopharyngeal cancer	FaDu	140 μM	Inhibition of proliferation; Induction of apoptosis	(108)

## 5 Limitations and future perspectives

Ginsenosides have been shown to regulate HIF-1α both directly and indirectly. Direct effects include the promotion of HIF-1α degradation and the inhibition of HIF-1α transcriptional activity. HIF-1α can directly transcribe from either of two transactivation domains, each of which is regulated by distinct mechanisms (110). Because N-terminal transactivation domain is located in the ODD structural domain, direct regulation by ginsenosides includes promoting HIF-1α degradation through the ubiquitin-proteasome pathway to enhance its protein expression (100). In addition, ginsenosides can promote hydroxylation of the C-terminal transactivation domain structural domain of HIF-1α, and regulate its transcriptional activity by interacting with coactivators such as CBP/P300 (111). Indirect regulation occurs through upstream signaling pathways, such as PI3K/Akt/mTOR, MAPK/ERK, and NF-κB, which influence HIF-1α stability, translation, and transcription. Additionally, pathways like Wnt/*β*-catenin and oxidative stress-mediated ROS signaling contribute to the modulation of HIF-1α activity (94). These mechanisms highlight the diverse roles of ginsenosides in regulating HIF-1α and suppressing tumor progression. Based on the summarized articles, ginsenosides, as HIF-1α inhibitors, can significantly suppress cancer cells through multiple dosing and local injection methods, exhibiting minimal drug toxicity during this process. With the expansion of pharmacological activity research of *P. ginseng* under hypoxic conditions, the use of *P. ginseng* in cancer treatment has made significant progress.

However, there are still some constraints to consider. The anti-cancer activity of ginsenosides correlates with the number of sugar moieties; as the number of sugar moieties in the ginsenoside molecule decreases, its anti-cancer activity increases. Therefore, ginsenosides containing four or more sugar molecules, such as Rb1 and Rc, exhibit no significant anticancer effects (112). In practical applications, the antitumor effects of ginsenosides are constrained by low bioavailability, limited membrane permeability, and a relatively short half-life *in vivo.* In recent years, highly efficient and safe nanocarrier delivery systems have been employed in cancer therapy (113, 114). These nanocarrier systems significantly enhance the solubility, stability, and bioavailability of ginsenosides while improving their tumor-targeted accumulation, endowing ginsenosides with tremendous potential for medical applications. A more comprehensive investigation of the active metabolites in *P. ginseng* can be conducted in future studies. Other components of *P. ginseng*, such as polysaccharides, also show potential therapeutic effects against cancer; however, their specific mechanisms related to HIF-1α pathways remain unclear. Although advances have been made in understanding the role of multiple ginsenosides in alleviating cancer progression through the HIF-1α pathway in hypoxic environments, there are a variety of other active metabolites in *P. ginseng* that still require further study. Therefore, future studies should focus on determining whether other active metabolites in *P. ginseng* can exert anti-cancer effects through the HIF-1α pathway, which could provide more opportunities for the clinical application of *P. ginseng*’s anti-cancer effects. Looking ahead, we will focus on the study of *P. ginseng* polysaccharides inhibiting glycolysis of tumor cells through the HIF-1α pathway and promoting apoptosis of cancer cells, to achieve the therapeutic effect on cancer.

## 6 Conclusion

HIF-1α targeting in evidence of the importance of ginsenosides as anticancer agents is mainly reflected in their mechanisms of action on the regulation of the TME, angiogenesis inhibition, metastasis inhibition, et al. HIF-1α is a core regulator of tumors adapting to the hypoxic microenvironment, especially in solid tumors, where hypoxia activates HIF-1α, which in turn regulates the expression of downstream target genes and promotes malignant tumor progression. Studies have shown that ginsenosides can block the downstream signaling pathway of HIF-1α by directly or indirectly inhibiting the activity of HIF-1α, including the inhibition of the stability of HIF-1α and the modulation of the upstream signaling pathway of HIF-1α. Through the inhibition of HIF-1α, ginsenosides can play an anticancer role at multiple levels, enhance drug efficacy, and inhibit the metastatic recurrence of cancer cells. The naturally low toxicity and synergistic potential of ginsenosides make them an important addition to future comprehensive clinical cancer therapies. In addition, current exploration of the anticancer effects of *P. ginseng* primarily focuses on ginsenosides, with less attention paid to other active metabolites such as polysaccharides. We acknowledge the promising potential of these components and propose that future research should further explore their roles in HIF-1α regulation. Therefore, the study of the therapeutic effects of the other active metabolites in the *P. ginseng* on cancer through HIF-1α offers broad research prospects.

## Glossary

HIF-1α: Hypoxia-inducible factor-1α
TME: tumor microenvironment
*P. ginseng*: *Panax ginseng* Meyer
TAD: trans-activating structural domains
N-TAD: N-terminal transactivation domain
C-TAD: C-terminal transactivation domain
ODD: oxygen-dependent degradation
HIF-1β: Hypoxia-inducible factor-1β
PHD: proline-4-hydroxylase
VHL: von Hippel–Lindau
FIH: factor inhibiting HIF-1
EGF: epidermal growth factor
EGFR: epidermal growth factor receptor
ERK1/2: extracellular signal-regulated kinase 1/2
HREs: hypoxia response elements
VEGFs: vascular endothelial growth factors
EMT: Epithelial-mesenchymal transition
EMT-TFs: EMT-inducing transcription factors
ZEB1: zinc finger E-box binding homeobox 1
TCA: tricarboxylic acid
NF-κB: nuclear factor-κB
ERK: extracellular signal-regulated kinase
PI3K: phosphatidylinositol 3-kinase
AKT: protein kinase B
mTOR: mammalian target of rapamycin
p70S6K: phospho-p70 S6 kinase
CA4P: Combretastatin A4 phosphate
MAPK: mitogen-activated protein kinase
C3DM: 3β-O-Glc-DM
PDQ: Pseudoginsengenin DQ
